# Identification of Fungal Pathogens by Visible Microarray System in Combination with Isothermal Gene Amplification

**DOI:** 10.1007/s11046-014-9756-2

**Published:** 2014-06-22

**Authors:** Kanae Sakai, Plinio Trabasso, Maria Luiza Moretti, Yuzuru Mikami, Katsuhiko Kamei, Tohru Gonoi

**Affiliations:** 1 Division of BioResources, Medical Mycology Research Center, Chiba University, Inohana 1-8-1, Chuo-ku, Chiba, Japan; 2 Departamento de Clínica Médica, Faculdade de Ciências Médicas, Universidade Estadual de Campinas, Campinas, SP Brazil; 3 JST/JICA, Science and Technology Research Partnership for Sustainable Development (SATREPS), Chiba, Japan

**Keywords:** Diagnosis, Microarray, Fungal infection, Isothermal amplification

## Abstract

The increasing incidence of infectious diseases caused by fungi in immunocompromised patients has encouraged researchers to develop rapid and accurate diagnosis methods. Identification of the causative fungal species is critical in deciding the appropriate treatment, but it is not easy to get satisfactory results due to the difficulty of fungal cultivation and morphological identification from clinical samples. In this study, we established a microarray system that can identify 42 species from 24 genera of clinically important fungal pathogens by using a chemical color reaction in the detection process. The array uses the internal transcribed spacer region of the rRNA gene for identification of fungal DNA at the species level. The specificity of this array was tested against a total of 355 target and nontarget fungal species. The fungal detection was succeeded directly from 10^3^ CFU/ml for whole blood samples, and 50 fg DNA per 1 ml of serum samples indicating that the array system we established is sensitive to identify infecting fungi from clinical sample. Furthermore, we conducted isothermal amplification in place of PCR amplification and labeling. The successful identification with PCR-amplified as well as isothermally amplified target genes demonstrated that our microarray system is an efficient and robust method for identifying a variety of fungal species in a sample.

**Electronic supplementary material:**

The online version of this article (doi:10.1007/s11046-014-9756-2) contains supplementary material, which is available to authorized users.

## Introduction

Systemic fungal infections with high morbidity and mortality rates in immunocompromised patients are growing. Besides the increasing incidence, recent epidemiology of fungal infection shows the expanding variety of fungal pathogens [1].

Identification of the causative pathogen is a fundamental step for appropriate treatment of infectious diseases, and early initiation of antifungal therapy is crucial for reducing the mortality rate in infected patients. Despite efforts by many researchers, however, early and rapid diagnosis of systemic fungal infection remains limited. Conventional diagnostic procedures, such as cultivation of fungi from clinical samples, are time-consuming and suffer from low sensitivity. Furthermore, sufficient technique and experience are required at the identification step. In recent years, other methods, such as PCR and serological tests, have been established for rapid and sensitive detection of fungi from clinical samples [2, 3]. However, these methods are difficult to use to identify a variety of fungal species at a time. Although multiplex PCR can be used to identify several species in one test, its applicability is limited by the primer sets used because specific primer sets are needed for each species.

A variety of DNA array systems have been developed to identify several bacteria and/or fungi simultaneously with high sensitivity and specificity [4–8]. Fluorescent labels are widely used to detect the signal in DNA microarrays due to their high sensitivity. However, the low stability of most fluorescent dyes and the necessity of expensive scanning equipment call for the development of alternative labeling systems that are inexpensive and robust.

To facilitate the diagnosis of fungal infectious disease, we established a rapid and specific DNA microarray system for identifying a variety of causative fungal species simultaneously. We applied the chemical color reaction of biotin-peroxidase and its substrate as the signal detection for the microarray system, enabling examination of the spot pattern with the naked eyes, without the need for expensive scanning equipment. To evaluate the specificity and sensitivity of this visible DNA microarray system, we tested it on several kinds of samples, such as reference fungal strains, blood samples containing a certain number of fungal cells, serum samples with serial dilutions of fungal DNA, and blood culture samples from patients.

Conventional PCR methods have been used for labeling and amplifying DNA from pathogen in microarray identification systems, but this method could not be used for bedside analysis and therefore difficult to be widely adopted. Recently, several isothermal amplification methods that do not require expensive thermal cyclers, such as loop-mediated isothermal amplification (LAMP), helicase-dependent amplification (HAD), and isothermal and chimeric primer-initiated amplification of nucleic acids (ICAN), have been developed to replace PCR amplification [9–12]. However, the maximum amplifiable length of the products in these isothermal methods is too small (100–500 bp) for our purposes, making primer design difficult. Hence, in this study, we applied the recombinase polymerase amplification cycle (RPA) for labeling and amplification of DNA products from the pathogen for microarray detection [13]. The RPA technology is based on a combination of polymerases and DNA recombinases. These enzyme mixtures are active at low temperature (optimum around 37 °C) and recognize template target sites by oligonucleotide primers, followed by strand-displacing DNA synthesis. Thus, exponential DNA amplification of the target region is proceeded under the isothermal condition.

## Materials and Methods

### Microorganisms and Growth Conditions

A total of 355 strains were obtained from the Medical Mycology Research Center IFM Collection (Chiba university, Japan) (Table S1). All fungal strains were cultivated on PDA medium at appropriate temperatures. Small amount of fungal cells were picked by toothpick and suspended in distilled water to become little bit cloudy solution and used as template for PCR amplification.

### Design of Capture Probes

The fungal oligonucleotide probes were designed based on the whole internal transcribed spacer (ITS) sequences regions available in the GenBank database and from our own sequencing data. The alignment was prepared by BioEdit using several objective and nonobjective fungal ITS sequences as listed in Table S2. After sequence alignment, species- or genus-specific oligonucleotide sequences were selected to be unique to each species/genus. To evaluate the specificities against other organisms, we performed additional BLASTN searches of the GenBank database. The designed probes were consisted of 14–21 species/genus-specific oligonucleotides and a poly-T anchor at the end of the oligonucleotides [14]. Detailed sequences of the capture probes are given in Table 1.

**Table 1:** Oligonucleotide sequence of probes

Organism	Probe name	Probe sequence (5′-3′)	Length (bp)
Common for all fungi	50-17	GATGAAGAACGCAGCGATTTTTTTTTT	27
	50-19	CGATGAAGAACGCAGCGAATTTTTTTTTT	29
	51-17	GAGTCTTTGAACGCACATTTTTTTTTT	27
	51-19	CGAGTCTTTGAACGCACATTTTTTTTTTT	29
	52-17R	TTTTTTTTTTACCAAGAGATCCGTTGT	27
	52-19R	TTTTTTTTTTAACCAAGAGATCCGTTGTT	29
*Absidia corymbifera*	Ab3-19t	CCGGATGGAGACTCTAGAGTTTTTTTTTT	29
	Ab2-18Rt	ATTTAAGGCCATGACAGCTTTTTTTTTT	28
	Ab2-t18R	TTTTTTTTTTATTTAAGGCCATGACAGC	28
*Alternaria* sp.	AlA-17Rt	GAAGTACGCAAAAGACATTTTTTTTTT	27
	AlA-t17R	TTTTTTTTTTGAAGTACGCAAAAGACA	27
	AlD-16Rt	ACGCCCAACACCAAGCTTTTTTTTTT	26
	AlE-16t	TCGGAGCGCAGCACAATTTTTTTTTT	26
*Aspergillus flavus*	60B 1	TTTTTTTTTTTGATCTAGTGAAGTCTGAG	29
	60B 1R	TTTTTTTTTTCTCAGACTTCACTAGATCA	29
	60B 17R	TCAGACTTCACTAGATCTTTTTTTTTT	27
	60C 1R	TTTTTTTTTTTAACTGATTGCGATACAAT	29
	60C 2R	TTTTTTTTTTACTGATTGCGATACAAT	27
	60C-19R	TAACTGATTGCGATACAATTTTTTTTTTT	29
*Aspergillus fumigatus*	33B-1R	TTTTTTTTTTTAACTGATTACGATAATCAA	30
	33B-2R	TTTTTTTTTTTAACTGATTACGATAATCA	29
	33B-4R	TAACTGATTACGATAATCAATTTTTTTTTT	30
	33C 1R	TTTTTTTTTTTAACTGATTACGATAATCAAC	31
	33C 2R	TTTTTTTTTTACTGATTACGATAATCAAC	29
	33C 3R	TTTTTTTTTTCTGATTACGATAATCAAC	28
	34A-8	TTGTCACCTGCTCTGTTTTTTTTTTT	26
	34A-14	TTGTCACCTGCTCTTTTTTTTTTT	24
	34A-17	GTCACCTGCTCTGTTTTTTTTTT	23
	34A-20	TTTTTTTTTTTTTGTCACCTGCTC	24
*Aspergillus nidulans*	64B 8	TTTTTTTTTTAGTTCAGTGGTCCCCGGC	28
	64B 9	TTTTTTTTTTAGTTCAGTGGTCCCCG	26
	65A 15	GGCGTCTCCAACCTTTTTTTTTTTT	25
	65A 17	CGGCGTCTCCAACCTTATTTTTTTTTT	27
	65A 19	CCGGCGTCTCCAACCTTATTTTTTTTTTT	29
*Aspergillus niger*	62A 4	TTTTTTTTTTATAGACACGGATG	23
	63A 15	TTTTTTTTTTCCAACCATTCTTTCCA	26
	63A 17	TTTTTTTTTTTCCAACCATTCTTTCCA	27
	63A 19	TTTTTTTTTTTTCCAACCATTCTTTCCAG	29
*Aspergillus terreus*	35A 17R	GCAAAGAATCACACTCATTTTTTTTTT	27
	35A 19	TGAGTGTGATTCTTTGCAATTTTTTTTTT	29
	35A 19R	TTGCAAAGAATCACACTCATTTTTTTTTT	29
	36A 1	TTTTTTTTTTGGCTTCGTCTTCCGCTCCG	29
	36A 2	TTTTTTTTTTGCTTCGTCTTCCGCTCC	27
	36A 19	GGCTTCGTCTTCCGCTCCGTTTTTTTTTT	29
	36B 15	CGACGCATTTATTTGTTTTTTTTTT	25
	36B 17	GCCGACGCATTTATTTGTTTTTTTTTT	27
	36B 19	CGCCGACGCATTTATTTGCTTTTTTTTTT	29
*Blastomyces dermatitidis*	41A 17R	GTTCCTCCGGTCTAGGATTTTTTTTTT	27
	41A 19R	GGTTCCTCCGGTCTAGGAGTTTTTTTTTT	29
	42A 15	CCGGCCCCATCTCAATTTTTTTTTT	25
	42A 17	TCCGGCCCCATCTCAAATTTTTTTTTT	27
*Candida albicans*	14A 15	CGGAGATGCTTGACTTTTTTTTTTT	25
	14A 17	CGGAGATGCTTGACAATTTTTTTTTTT	27
	1A 17R	TTTTTTTTTTAAGTTTAGACCTCTGGC	27
	1A 19	CCGCCAGAGGTCTAAACTTTTTTTTTTTT	29
	1B 15R	TTTTTTTTTTATCTGGTGTGACAAG	25
	1B 17R	TTTTTTTTTTTAATCTGGTGTGACAAG	27
	1B 19	ACTTGTCACACCAGATTATTTTTTTTTTT	29
	2A 15	CGTCCACCACGTATATTTTTTTTTT	25
	2A 17	AACGTCCACCACGTATATTTTTTTTTT	27
	2A 19	GTAACGTCCACCACGTATATTTTTTTTTT	29
	2B 15	TTTTTTTTTTATTGCTTGCGGCGGT	25
	2B 17	TTTTTTTTTTACATTGCTTGCGGCGGT	27
*Candida dubliniensis*	13A-2R	TTTTTTTTTTAACAAAACACATGTGGT	27
	13A-3R	TTTTTTTTTTAACAAAACACATGTGG	26
	13B 15	TTTTTTTTTTTATAAACTTGTCACG	25
	13B 17	TTTTTTTTTTTATAAACTTGTCACGAG	27
*Candida famata*	80B-1	TGGTCTGGACTAGAAATATTTTTTTTT	28
	80B-1R	TTTTTTTTTTATTTCTAGTCCAGACCA	28
	81A-1	TTTTTTTTTTTAGTGCTATATGACTTTC	28
	81A-3	TTTTTTTTTTAGTGCTATATGACTTTC	27
*Candida glabrata*	7A 15R	TTTTTTTTTTTGTCTCTCTCCGAGC	25
	7A 17R	TTTTTTTTTTATGTCTCTCTCCGAGCT	27
	7A 19R	TTTTTTTTTTGATGTCTCTCTCCGAGCTC	29
	7B 17	CTCCTGCCTGCGCTTAATTTTTTTTTT	27
	7B 19	TTCTCCTGCCTGCGCTTAATTTTTTTTTT	29
	7B 19R	TTAAGCGCAGGCAGGAGAATTTTTTTTTT	29
	8A 17	TTTTTTTTTTAACTTGAAATTGTAGGC	27
	8A 19	TTTTTTTTTTAACTTGAAATTGTAGGCCA	29
	8B 15	TTTTTTTTTTTGCTGCTCGTTTGCG	25
	8B 17	TTTTTTTTTTTTGCTGCTCGTTTGCGC	27
	8B 19	TTTTTTTTTTTCTGCTGCTCGTTTGCGCG	29
*Candida guilliermondii*	55A 17R	TTTTTTTTTTAAAATTTGACTAACTGT	27
	55A 19	TTTACAGTTAGTCAAATTTTTTTTTTTTT	29
	55A 19R	TTTTTTTTTTCAAAATTTGACTAACTGTA	29
	55B 15	GTCGACCTCTCAATGTTTTTTTTTT	25
	55B 17	TGTCGACCTCTCAATGTTTTTTTTTTT	27
	55B 19	CTGTCGACCTCTCAATGTATTTTTTTTTT	29
*Candida kefyr*	Ck1-t16R	TTTTTTTTTTGTCAGACGATTCCCCC	26
	Ck2-20Rt	TAGCAGAGAATCAAGAACTGTTTTTTTTTT	30
	Ck2-t20R	TTTTTTTTTTTAGCAGAGAATCAAGAACTG	30
	Ck4-t17	TTTTTTTTTTCGTCTCGGGTTAACTTG	27
	Ck4-17Rt	CAAGTTAACCCGAGACGTTTTTTTTTT	27
	Ck4-t17R	TTTTTTTTTTCAAGTTAACCCGAGACG	27
	Ck6-18Rt	GCAAGAGTCGAGTCCATATTTTTTTTTT	28
*Candida krusei*	9B 17R	GCTATATTCCACATTTTTTTTTTTTTT	27
	9B 19R	ATGCTATATTCCACATTTTTTTTTTTTTT	29
	9C-1R	TTTTTTTTTTTCGACTATATGCTATATTC	29
	9C-2R	CGACTATATGCTATATTCCTTTTTTTTTT	29
	9C-3R	TTTTTTTTTTCGACTATATGCTATATTCC	29
	10A 15	GCGGACGACGTGTAATTTTTTTTTT	25
	10A 17	GCGGACGACGTGTAAAGTTTTTTTTTT	27
	10A 19	GAGCGGACGACGTGTAAAGTTTTTTTTTT	29
	10B 15	TTTTTTTTTTGAGCGAAGCTGGCCG	25
	10B 17	TTTTTTTTTTAGCGAAGCTGGCCGAGC	27
	10B 19	TTTTTTTTTTGAGCGAAGCTGGCCGAGCG	29
*Candida lusitaniae*	11C 14R	TGTTCGCAAAAACATTTTTTTTTT	24
	11C 15R	TGTTCGCAAAAACAATTTTTTTTTT	25
	11C 16R	TGTTCGCAAAAACAATTTTTTTTTTT	26
	11B 19	TTCGAATTTCTTAATATCATTTTTTTTTT	29
	11B 19R	TTGATATTAAGAAATTCGATTTTTTTTTT	29
	12A 17R	TTTCGGAGCAACGCCTATTTTTTTTTT	27
	12A 19	TTAGGCGTTGCTCCGAAATTTTTTTTTT	29
	12A 19R	TTTCGGAGCAACGCCTAACTTTTTTTTTT	29
	12B 17	CGTTTACAGCACGACATTTTTTTTTTT	27
	12B 19	CACGTTTACAGCACGACATTTTTTTTTTT	29
*Candida rugosa*	17B 15R	GATCGGTACTTGAAGTTTTTTTTTT	25
	18B 15R	TTTTTTTTTTAGACGGTCGCGTTTC	25
*Candida parapsilosis*	5A 17	CTGCCAGAGATTAAACTTTTTTTTTTT	27
	5A 18	CTGCCAGAGATTAAACTCTTTTTTTTTT	28
	5A 18R	GAGTTTAATCTCTGGCAGTTTTTTTTTT	28
	6A 17	TTTTTTTTTTCCAAAACTTCTTCCATT	27
	6A 19	TTTTTTTTTTCTCCAAAACTTCTTCCATT	29
	6A 19R	TTTTTTTTTTAATGGAAGAAGTTTTGGAG	29
	6B 17	TTTTTTTTTTACTCCAAAACTTCTTCC	27
	6B 18	TTTTTTTTTTACTCCAAAACTTCTTCCA	28
	6B 18R	TTTTTTTTTTTGGAAGAAGTTTTGGAGT	28
*Candida tropicalis*	3A 16R	TTTTTTTTTTGGATTGCTCCCGCCAC	26
	3A 17R	TTTTTTTTTTGGATTGCTCCCGCCACC	27
	3B 15R	TTTTTTTTTTATCAAGTTTGACTGT	25
	3B 17R	TTTTTTTTTTAAATCAAGTTTGACTGT	27
	3B 19R	TTTTTTTTTTAAATCAAGTTTGACTGTAA	29
	4A 15	TTTTTTTTTTATACGCTAGGTTTGT	25
	4A 17	TTTTTTTTTTATACGCTAGGTTTGTTT	27
	4A 19	TTTTTTTTTTCAATACGCTAGGTTTGTTT	29
	4B 17	GCTAGTGGCCACCACTTTTTTTTTTTT	27
	4B 19	GCTAGTGGCCACCACAATTTTTTTTTTTT	29
*Candida zeylanoides*	15A 19	GTTTTATACTAAAACTTCATTTTTTTTTT	29
	15A 1	GGTTTTATACTAAAACTTCATTTTTTTTTT	30
	15B 1	TTTTTTTTTTATTGAATTGTTAATTAATTA	30
	15B 1R	TTTTTTTTTTTAATTAATTAACAATTCAAT	30
	16A 19	TTTTTTTTTTGACCAGTATAGTATTTGT	28
	16A 17	TTTTTTTTTTACCAGTATAGTATTTG	26
*Coccidioides posadasii*	37C 15R	GGAGGTGCGCAGCCGTTTTTTTTTT	25
	37C 17R	GGGAGGTGCGCAGCCGGTTTTTTTTTT	27
	37C 19R	GGGGAGGTGCGCAGCCGGATTTTTTTTTT	29
	37E 15R	TTTTTGCTATGATGCTTTTTTTTTT	25
	37E 17R	GATTTTTGCTATGATGCTTTTTTTTTT	27
	37E 18R	GATTTTTGCTATGATGCTTTTTTTTTTT	28
	38D-1	TTATATCCGGTTTGACCTCTTTTTTTTTT	29
	38D-2	ATATCCGGTTTGACCTCTTTTTTTTTT	27
	38D-3	TATCCGGTTTGACCTTTTTTTTTTT	25
	38E 15	TTTTTTTTTTACCCGATCGGGGCCG	25
	38E 17	TTTTTTTTTTGACCCGATCGGGGCCGA	27
	38E 19	TTTTTTTTTTAGACCCGATCGGGGCCGAT	29
*Cryptococcus neoformans var. neoformans, grubii, gattii*	22A-8	GTTTATGTGCTTCGGCACTTTTTTTTTT	28
	22A 17	TTTTTTTTTTGTTTATGTGCTTCGGCA	27
	23A 17	TTTTTTTTTTGAAGGTGATTACCTGTC	27
	23A 19	TTTTTTTTTTGGAAGGTGATTACCTGTCA	29
	23B 1	TTTTTTTTTTTTTCGCTGGGCCTATGG	27
	23B 2	TTTTTTTTTTGTTTCGCTGGGCCTATGGG	29
*Cryptococcus gattii*	20-2R	TTTTTTTTTTTGGACCGAAGCCCAGTATT	29
	20-5R	TTTTTTTTTTGACCGAAGCCCAGTATT	27
	20-6R	TTTTTTTTTTGGACCGAAGCCCAGTAT	27
*Cunninghamella bertholletiae*	70A-1R	CCCAAAGATCCCTTGATCTATTTTTTTTTT	30
	70A-2R	CCCAAAGATCCCTTGATCTTTTTTTTTTT	29
	71A 19	TAGTCGGCTTTAATAGATTTTTTTTTTTT	29
	71A 17	TAGTCGGCTTTAATAGATTTTTTTTTT	27
	71A 15	AGTCGGCTTTAATAGTTTTTTTTTT	25
	71B-1	TTTTTTTTTTTAAATACAAGGCTCGACTTT	30
	71B-2	TTTTTTTTTTAATACAAGGCTCGACT	26
	71B-3	TTTTTTTTTTTAATACAAGGCTCGACTTT	29
*Epidermophyton floccosum*	76B 19R	CTCAGACTGAACCACCTATTTTTTTTTTT	29
	76B 17R	TCAGACTGAACCACCTATTTTTTTTTT	27
	76B 15R	CAGACTGAACCACCTTTTTTTTTTT	25
	77A 19	TTTTTTTTTTAGTTTCCGTCGGGAGGACG	29
	77A 17	TTTTTTTTTTGTTTCCGTCGGGAGGAC	27
*Fusarium* sp.	7-16t	GGCCACGCCGTTAAACTTTTTTTTTT	26
	7-18t	CTTCTGAATGTTGACCTCTTTTTTTTTT	28
	7-19t	CGCGGCCACGCCGTTAAACTTTTTTTTTT	29
	7B-19t	CAACTTCTGAATGTTGACCTTTTTTTTTT	29
	7C-18t	ACCCCAACTTCTGAATGTTTTTTTTTT	28
	7C-19t	CCGTAAACCCCAACTTCTGTTTTTTTTTT	29
	10B-16Rt	GTATGTTCACAGGGGTTTTTTTTTTT	26
	10B-18Rt	GTATGTTCACAGGGGTTGTTTTTTTTTT	28
*Fusarium solani* complex (FSSC)	1-16Rt	CCGTCTGTTCCCGCCGTTTTTTTTTT	26
	1-18Rt	GCCGTCTGTTCCCGCCGATTTTTTTTTT	28
	1-19Rt	CCGTCTGTTCCCGCCGAAGTTTTTTTTTT	29
	2-19Rt	GCCGATCCCCAACGCCAGGTTTTTTTTTT	29
	4-18t	CACCTCGCAACTGGAGAGTTTTTTTTTT	28
	4-19t	GCTAACACCTCGCAACTGGATTTTTTTTTT	29
	4-20t	GTAGCTAACACCTCGCAACTTTTTTTTTTT	30
	6B-17Rt	CAGAGTTAGGGGTCCTCTTTTTTTTTT	27
	9-17t	ACGTTGCTTCGGCGGGATTTTTTTTTT	27
*Histoplasma capsulatum*	39B-22	TTTTTTTTTTCGTTCACCGACGGTTCTT	28
	39B-24	TTTTTTTTTTGTTCACCGACGGTTCT	26
	39B-25	TTTTTTTTTTGTTCACCGACGGTTC	25
	39C 15R	AGGTCCGGTAGACAATTTTTTTTTT	25
	39C 17R	CAGGTCCGGTAGACAAGTTTTTTTTTT	27
	39C 19R	ACAGGTCCGGTAGACAAGGTTTTTTTTTT	29
*Malassezia furfur*	48A 15R	TTTTTTTTTTCCAAACGGTGCACAC	25
	48A 17R	TTTTTTTTTTTCCAAACGGTGCACACG	27
	48A 19R	GATTTCCACGTTCATACAATTTTTTTTTT	29
	48B 15R	TTTCCACGTTCATACTTTTTTTTTT	25
	48B 17R	ATTTCCACGTTCATACATTTTTTTTTT	27
	48B 19R	GATTTCCACGTTCATACAATTTTTTTTTT	29
	49A 7	TGCGATTGCACTGCTTTGTTTTTTTTTT	28
	49A 8	GCGATTGCACTGCTTTGTTTTTTTTTT	27
	49A 9	CGATTGCACTGCTTTGTTTTTTTTTT	26
	49B 15	TTTTTTTTTTGCATTAGCGCCTTTG	25
	49B 17	TTTTTTTTTTTGCATTAGCGCCTTTGG	27
	49B 19	TTTTTTTTTTATGCATTAGCGCCTTTGGG	29
*Microsporum canis*	73A 6	TTTTTTTTTTGTAACCACCCACCGCTTA	28
	73A 7	GTAACCACCCACCGCTTAGTTTTTTTTTT	29
	73A 9	GTAACCACCCACCGCTTATTTTTTTTTT	28
	73B 19	CGCACCATGTATTATTCAGTTTTTTTTTT	29
	73B 17	GCACCATGTATTATTCATTTTTTTTTT	27
	73B 1	TTTTTTTTTTCGCACCATGTATTATTCAG	29
Microsporum gypseum	74A 2R	GATTTTACTTGCTAACGTTTTTTTTTT	27
	74B 1	CGGAACAGTATTCATGGATTTTTTTTTTT	29
	74B 2	GGAACAGTATTCATGGATTTTTTTTTT	27
	74B 4	TTTTTTTTTTCGGAACAGTATTCATGGAT	29
*Mucor* sp.	M1-t15R	TTTTTTTTTTTAATACAGTTCACAG	25
	M1-16Rt	AATAATACAGTTCACATTTTTTTTTT	26
	M1-t16R	TTTTTTTTTTAATAATACAGTTCACA	26
	M3-20Rt	GGTAAATAATAATAGGATACTTTTTTTTTT	30
	M3-t20R	TTTTTTTTTTGGTAAATAATAATAGGATAC	30
	M4-t15R	TTTTTTTTTTGGTCTATGTTACAAT	25
*Paracoccidioides brasiliensis*	45A 15R	CCCCGTCCCCCCACGTTTTTTTTTT	25
	45A 17R	GCCCCGTCCCCCCACGGTTTTTTTTTT	27
	45A 18R	GGCCCCGTCCCCCCACGGTTTTTTTTTT	28
	45B 15R	TTTTTTTTTTTCAAAGCTCCGAACC	25
	45B 17R	TTTTTTTTTTGTCAAAGCTCCGAACCA	27
	45B 19R	TTTTTTTTTTCGTCAAAGCTCCGAACCAG	29
	46A 15	CCCCACTCATCGACCTTTTTTTTTT	25
	46A 17	GCCCCACTCATCGACCCTTTTTTTTTT	27
	46A 19	GGCCCCACTCATCGACCCCTTTTTTTTTT	29
*Penicillium marneffei*	43B 15R	TTTTTTTTTTTCAGACAGTCCATCT	25
	43B 17R	TTTTTTTTTTCTCAGACAGTCCATCTT	27
	43B 19R	TTTTTTTTTTACTCAGACAGTCCATCTTC	29
	44A 17	TTTTTTTTTTCCACCATATTTACCACG	27
	44A 19	TTTTTTTTTTACCACCATATTTACCACGG	29
*Pichia anomala*	Pa2-16Rt	GACTATTGGTTAAAGGTTTTTTTTTT	26
	Pa3-17t	AGCAGTCTTTCTGAAATTTTTTTTTT	27
	Pa3-t17	TTTTTTTTTTAGCAGTCTTTCTGAAAT	27
	Pa4-20Rt	CTTCTAAACCTGCCTAGCTGTTTTTTTTTT	30
	Pa4-t20R	TTTTTTTTTTCTTCTAAACCTGCCTAGCTG	30
*Pichia norvegensis*	Pin2-20t	CACGAATAACCATGTCACCCTTTTTTTTTT	30
	Pin2-t20	TTTTTTTTTTCACGAATAACCATGTCACCC	30
	Pin2-20Rt	GGGTGACATGGTTATTCGTGTTTTTTTTTT	30
	Pin2-t20R	TTTTTTTTTTGGGTGACATGGTTATTCGTG	30
	Pin4-17t	GGCAGCGGGACTGAGCGTTTTTTTTTT	27
	Pin4-t17	TTTTTTTTTTGGCAGCGGGACTGAGCG	27
	Pin4-t17R	TTTTTTTTTTCGCTCAGTCCCGCTGCC	27
	Pin5-20t	CACTCGCGCTTGGCCCGCCGTTTTTTTTTT	30
	Pin5-t20	TTTTTTTTTTCACTCGCGCTTGGCCCGCCG	30
	Pin5-20Rt	CGGCGGGCCAAGCGCGAGTGTTTTTTTTTT	30
*Rhizomucor* sp.	Rm1-17t	AGGGATTGCTCCAGATCTTTTTTTTTT	27
	Rm1-t17R	TTTTTTTTTTGATCTGGAGCAATCCCT	27
	Rm2-17t	CTTTGGATTTGCGGTGCTTTTTTTTTT	27
	Rm2-17Rt	GCACCGCAAATCCAAAGTTTTTTTTTT	27
	Rm3-19t	GGGCTTGCTTGGTATCTATTTTTTTTTT	29
	Rm3-19Rt	TAGATACCAAGCAAGCCCTTTTTTTTTT	29
	Rm4-19t	GATCTGAACTTAGACGGGATTTTTTTTTT	29
	Rm4-t19R	TTTTTTTTTTTCCCGTCTAAGTTCAGATC	29
Rhizopus microspores*	Rizm1-19Rt	CTGAGAAGTAAATCCCAGTTTTTTTTTT	29
	Rizm1-t19R	TTTTTTTTTTCTGAGAAGTAAATCCCAGT	29
	Rizm2-t20	TTTTTTTTTTCTGGCGATGAAGGTCGTAAC	30
	Rizm2-20Rt	GTTACGACCTTCATCGCCAGTTTTTTTTTT	30
	Rizm2-t20R	TTTTTTTTTTGTTACGACCTTCATCGCCAG	30
	Rizm3-19t	CTTCCTTGGGAAGGAAGGTTTTTTTTTT	29
	Rizm3-t19	TTTTTTTTTTCTTCCTTTGGGAAGGAAGG	29
	Rizm3-19Rt	CCTTCCTTCCCAAAGGAAGTTTTTTTTTT	29
	Rizm4B-17Rt	GCACGATGGCTAGGTAGTTTTTTTTTT	27
	Rizm4B-t17R	TTTTTTTTTTGCACGATGGCTAGGTAG	27
*Rhizopus oryzae*	Rizo1-19Rt	TACCCCAGAGGAAACCCTATTTTTTTTTT	29
	Rizo1-t19R	TTTTTTTTTTTACCCCAGAGGAAACCCTA	29
	Rizo2-t18R	TTTTTTTTTTCTCCTGAAACCAGGAGTG	28
	Rizo3A-19t	ACAGTGAGCACCTAAAATGTTTTTTTTTT	29
	Rizo3A-t19	TTTTTTTTTTACAGTGAGCACCTAAAATG	29
	Rizo3B-19t	GCTAGGCAGGAATATTACGTTTTTTTTTT	29
	Rizo3B-t19	TTTTTTTTTTGCTAGGCAGGAATATTACG	29
*Rhodotorula mucilaginosa*	Rho2-19Rt	CACCTCCTTCAATCATTAAGTTTTTTTTTT	29
	Rho2-t19R	TTTTTTTTTTCACCTCTTCAATCATTAAG	29
	Rho5-18Rt	CTAGACCGTAAAGGCCAGTTTTTTTTTT	28
	Rho5-17Rt	CGAGCTAGACCGTAAAGTTTTTTTTTT	27
	Rho5-t17R	TTTTTTTTTTCGAGCTAGACCGTAAAG	27
*Scedosporium prolificans*	Scp2-t15R	TTTTTTTTTTGTATTGTATTCAGAA	25
	ScpP-19Rt	GGCTTGTAAAAACCTAGGCTTTTTTTTTT	29
	ScP-t19R	TTTTTTTTTTGGCTTGTAAAAACCTAGGC	29
Sporothrix schenckii*	Sps2-t20R	TTTTTTTTTTGTAGGGCCCGCCGCCCCTGG	30
	Sps4-20t	CACAACTCCCAACCCTTGCTTTTTTTTTT	30
	Sps4-20Rt	GCAAGGGTTGGGAGTTGTGTTTTTTTTTT	30
	Sps4-17t	GCGAACCGTACCCAATCTTTTTTTTTT	27
*Trichophyton mentagrophytes*	68A 1	TTTTTTTTTTGTTTAGCCACTAAAGAGAG	29
	68A 2	TTTTTTTTTTGTTTAGCCACTAAAGAGA	28
	68A 4R	TTTTTTTTTTGTTTAGCCACTAAAGAGAGG	30
	69A-10	GCCCCCGTCTTTGGGGGTTTTTTTTTTT	28
*Trichophyton rubrum*	66B 6R	TTTTTTTTTTGCTCGAGGCTCCCAGAAGG	29
	66B 13R	TTTTTTTTTTCTCGAGGCTCCCAGAAGG	28
	66B 14R	TTTTTTTTTTGCTCGAGGCTCCCAGAAG	28
	67A 1	TTTTTTTTTTCAGCCAATCCAGCGCCCTCA	30
	67A 7	TTTTTTTTTTCAGCCAATCCAGCGCCCTC	29
	67A 8	TTTTTTTTTTAGCCAATCCAGCGCCCTCA	29
	67B 17	AGCCAATTCAGCGCCCTTTTTTTTTTT	27
	67B 19	CAGCCAATTCAGCGCCCTCTTTTTTTTTT	29
*Trichophyton tonsurans*	47A 6	CCTATCCTGGGGGGCCTTTTTTTTTT	26
	47A 7	TTTTTTTTTTCCTATCCTGGGGGGCC	26
	47A 19R	TTTTTTTTTTTATCCTGGGGGGCCGGCCT	29
	47B 1	TTTTTTTTTTGAGCCGCTATAAAGAGAGG	29
	47B 4	TTTTTTTTTTGAGCCGCTATAAAGAGAGGC	30
	47B 19R	GAGCCGCTATAAAGAGAGGTTTTTTTTTT	29
*Trichosporon* sp.	78A-3	TTTTTTTTTTCTTGCGCTCTCTGGTA	26
	78C-1	TTTTTTTTTTGCTCGCCTTAAAAGAGTT	28
*Trichosporon asahii*	79A-5	TTTTTTTTTTGCGTCTGCGATTTCT	25
	79A-6a	TTTTTTTTTTGGGCGTCTGCAATTTC	26
*Trichosporon cutaneum*	31A-2	TTTTTTTTTTCGGTCAATTGATTTTACAAA	30
	31A-4R	TTTGTAAAATCAATTGACCGTTTTTTTTTT	30
	32A 17	TTTTTTTTTTAACTTGTCTTATCTGGC	27

The probes with asterisk (*) shows cross-hybridization within the same genus

The ex-type classification name was used in some of the fungi

### Preparation of DNA Microarray Slides

The synthetic oligonucleotides were diluted to 20 pmol/μl in TE buffer and mixed with an equal volume of 6× SSC [20× SSC is 3 M NaCl, 0.3 M sodium citrate (pH7.0)] to make a final concentration of 10 pmol/μl oligonucleotides in 3× SSC. The probe solutions were spotted on NGK plastic slides (NGK insulators LTD, Aichi, Japan) using a KCS-mini microarray printer (Kubota Comps Corporation, Hyogo, Japan). After spotting, the slides were irradiated with UV at 0.6 J/cm^2^ using a UV cross-linker (model CL-1000; UVP, San Gabriel, Ca) to fix the probes on plastic slides. The slides were then gently shaken in blocking buffer [3 % BSA, 0.2 M NaCl, 0.1 M Tris–HCl (pH 8.0), and 0.05 % Triton X-100] for 5 min and washed with TE buffer for 10 min. The array slides were air-dried and stably stored at room temperature at least 3 years.

### Infectious Mouse Model

As an infection model, male ICR mice (Charles River Laboratories) were infected intravenously with *Aspergillus fumigatus* Af293 or *Fusarium solani* complex IFM40718 (FSSC) conidia (1 × 10^6^ conidia/mouse) in a 200 µl volume of saline. Three mice were used in each fungal species. One hour after infection, mice were killed, and blood was collected from the heart tissues under sterilized conditions. The CFU was determined by inoculating 100 µl of collected blood on a PDA with Chloramphenicol plate, and colonies were counted after 24 h of cultivation at 30 °C. Blood samples were used directly as template for PCR.

### Blood Culture

As the routine diagnosis of blood infection, blood samples were taken from patients and cultivated using the BD BACTEC FX system (BD, Tokyo, Japan) for 7 days, following the ethics of Chiba University Hospital. After cultivation, growth positive samples were inoculated onto several kinds of agar to identify bacteria and/or fungi. For the microarray identification, one growth positive and one growth negative blood culture samples were used directly as a template for PCR.

### DNA Extraction

Fungal DNA was extracted as normal phenol–chloroform method. The conidia of *A. fumigatus* were inoculated in PDB medium and cultivated for 2 days at 37 °C. The mycelium was collected by filtration and ground by mortar using liquid N_2_. The ground cells were suspended in DNA extraction buffer (200 mM Tris, 25 mM NaCl, 25 mM EDTA, 0.5 % SDS, pH 8.5) and extracted with phenol/chloroform/isoamyl alcohol. After that, RNase treatment and ethanol precipitation were conducted. The cells of *Candida albicans* were cultivated in PDB medium 1 day at 30 °C. The cells were collected by centrifugation, and the DNA was extracted using GenTorukun (TaKaRa Bio Inc., Shiga, Japan).

### PCR Amplification and Labeling

The 5′-biotin-labeled fungus-specific universal primers ITS1-bio (5′-TCCGTAGGTGAACCTGCGG-3′) and ITS4-bio (5′-TCCTCCGCTTATTGATATGC-3′) [15] were used for amplifying the entire ITS region and biotin labeling. The amplified fragment ranged from 426 to 930 bp depending on the fungal species. PCR was performed using MightyAmp DNA polymerase ver. 2 (TaKaRa Bio Inc.) in a total reaction volume of 10 μl containing 1 μl of template (fungal cell suspension, whole blood, serum, blood culture, and extracted DNAs). Amplification was carried out as follows, 2 min of initial denaturation at 98 °C, 40 cycles of DNA denaturation at 98 °C for 10 s, primer annealing at 55 °C for 15 s and elongation at 68 °C for 45 s, and a final elongation step at 68 °C for 5 min. After the PCR reaction, amplification was verified by electrophoresis. In case of low content of fungal cells or DNA, nested PCR was performed using ITS1-n (5′-GAGGAAGTAAAAGTCG-3′) and ITS4-n (5′-TTCACTCGCCGTTACT-3′) as the first-round PCR primer set. One µl of first-round PCR sample was then used as template in the second round of PCR performed in a total reaction volume of 10 μl.

### Isothermal Amplification

Isothermal amplification was performed with a TwistAmp basic kit (TwistDx Limited, Cambridge, UK). Amplification was carried out at 37 °C for 40 min according to the manufacturer’s protocol using 1.5 μl of fungal cell suspension or DNA as template.

### Microarray Hybridization and Signal Detection

Before microarray hybridization, amplified and labeled PCR samples were denatured at 95 °C for 2 min and chilled on ice for 2 min. Four μl of a denatured sample was then mixed with 16 μl of hybridization buffer (0.2 g tetramethylammonium chloride, 0.5 % SDS, and 1.9 mg EDTA in 1 ml of 6 × SSC). Samples were applied to the array slide, covered with a cover-film to prevent sample evaporation, and incubated at 37 °C for 1 h in a moist-chamber. Array slides were then washed with PBS buffer at 37 °C for 5 min. A color development reaction was performed on the slide in accordance with the avidin-biotinylated peroxidase complex (ABC) method using a 3, 3′, 5, 5′-tetramethylbenzidine (TMB) solution for visualization [16]. First, the conjugation reaction was performed with streptavidin and biotin-HRP for 30 min, and the array was washed twice with PBST buffer (PBS buffer with 0.1 % Tween 20) for 5 min. After washing, color development was performed with 0.02 % TMB, 0.015 % H_2_O_2_, and 0.5 mg/ml alginic acid in 0.2 M acetate buffer (pH 3.3). Color development was terminated after 30 min by washing the array slides with distilled water. The results were evaluated by visual observation.

## Results

### Design of Probes and DNA Microarray Slides

Because the ribosomal RNA gene, especially the 28S rRNA gene, is highly conserved among species, sequences of the ITS region are widely used for identification of fungal species. We designed species- and/or genus-specific probes within the ITS region and tested the specificity of selected sequences using 355 reference strains (Table S1). Genus-specific probes were designed for some fungi (*Alternaria* sp., *Rhizomucor* sp., *Mucor* sp., *Trichosporon* sp.); because they have highly conserved ITS region sequences within the genus, we could not design species-specific probes. We successfully designed 319 probes of species/genus-specific oligonucleotides ranging from 13 to 21 bp with a poly-T anchor at the 5′ or 3′ end for the identification of 42 species from 24 genera of fungal pathogens (Table 1). Three to twelve different specific capture probes were designed and spotted on the array slides for each fungal species/genus to ensure hybridization reaction for proper identification. Among the 319 probes, six universal probes for fungi were designed, so that the array would give a positive signal at universal probe even if fungal species in the tested sample was not listed on the Table 1. In other words, 6 universal probes could detect any fungi other than listed objective fungi without specific signal.

**Fig. 1:**
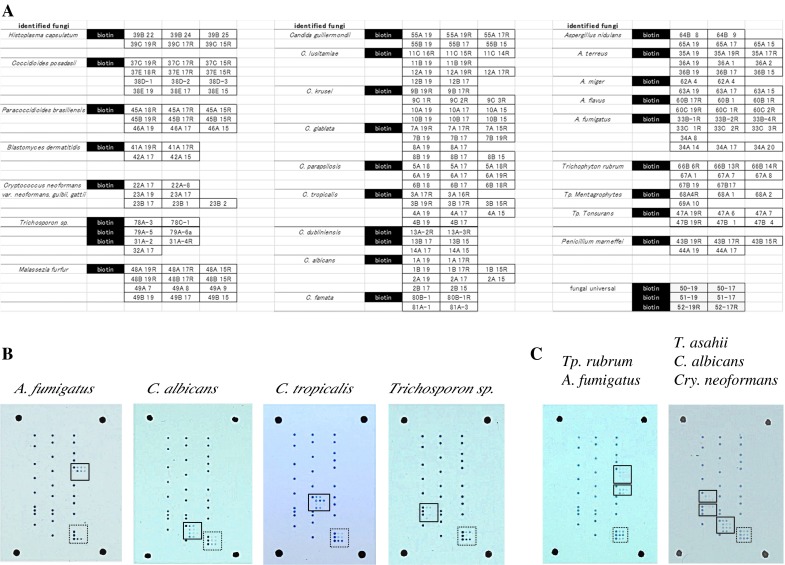
**a** Example layout of capture probes on microarray slide. Probe names correspond to probe names listed in Table 1. The *black column* labeled “biotin” indicates the spots for positive control (biotinylated-poly-T) and positional marker. **b** Typical hybridization patterns using fungal suspension of different fungal species as PCR template. Species-specific signals are enclosed in *solid line frames*, while universal signals for fungi are enclosed in *dotted line frames*. These figures show representative results for *A. fumigatus*, *Trichosporon asahii*, *C. tropicalis*, and *C. albicans*. **c** Simultaneous hybridization of several species in one array slide. Fungal cell mixtures of *C. albicans*, *Cryptococcus neoformans*, and *T. asahii* or of *A. fumigatus* and *Trichophyton rubrum* were directly used as template for PCR amplification and detected on the array slide

All designed probes and the positive control marker (biotinylated-poly-T) were spotted on one plastic slide. Figure 1a shows an example of the spot pattern of the microarray slide.

### Evaluation of the Specificity of DNA Microarray Probes

To evaluate the specificity of the designed capture probes, 66 fungal strains were used (Table S1). All fungal samples tested showed the expected species/genus-specific hybridization patterns as shown in Fig. 1b. Although some probes showed cross-hybridization within the same genus because of their highly conserved sequence (e.g., *Rhizopus stlonifer* was cross-hybridized to *Rhizopus microsporus* probes), listed organisms are the major fungi causing infection (Table 1). Moreover, the array system enabled us to identify all the mixed fungi in one test even when several fungal mixtures were used as template (Fig. 1c). Resulted spot number is sometimes varied depending on the sample (e.g., the spot of fungal common probes in Fig. 1b), because the affinity of each designed probes is different.

### Sensitivity of the DNA Microarray System

**Fig. 2:**
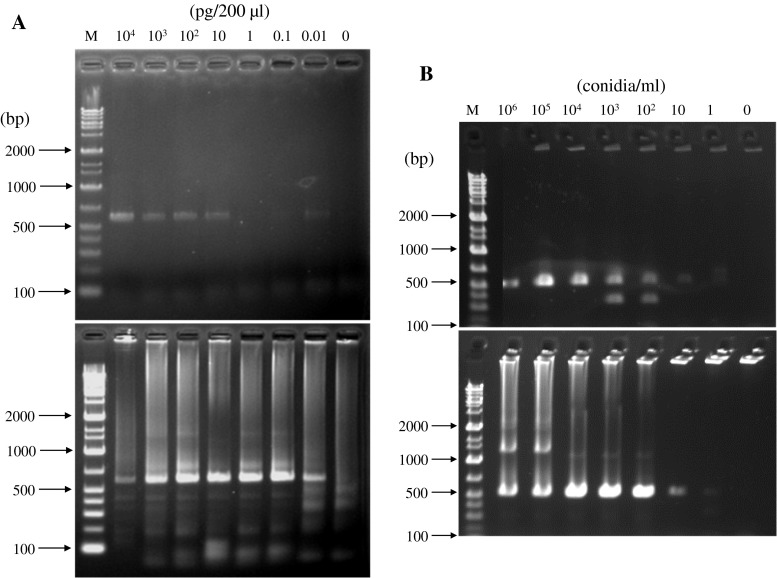
Agarose gel electrophoresis of PCR products. **a** PCR amplification of ITS region using blood sample spiked with *A. fumigatus* cells as template. *Upper panel* shows the results of normal PCR; *lower panel* shows the results of nested PCR. *Lanes*: M, molecular marker (Gene Ladder Wide 1: NIPPON GENE co., Tokyo, Japan); 1–7, blood sample spiked with conidia. **b** PCR amplification of ITS region using serum sample with *C. albicans* DNA as template. *Upper panel* shows the results of normal PCR; *lower panel* shows the results of nested PCR. *Lanes*: M, molecular marker; 1–8, DNA in 1 ml of serum

To evaluate microarray detection sensitivity, we used blood samples containing a known number of fungal cells and serum with fungal DNA in place of actual clinical samples. Serial ten-fold dilutions of fungal cells in blood (10^6^–10^0^ CFU/ml) were prepared by adding *A. fumigatus* conidia or *C. albicans* cells to rabbit whole blood. The PCR reaction was performed directly using 1 μl of rabbit whole blood with or without fungal cells as template (see Materials and Methods). After the PCR reaction, amplification was verified by electrophoresis, and the samples were used for microarray analysis (Fig. 2a). For both *A. fumigatus* and *C. albicans*, 10^3^ CFU/ml was the minimum concentration needed for PCR amplification followed by the microarray detection. Although 10^2^ CFU/ml could be considered as the limit of detection, amplification at this concentration is not reproducible. To increase the sensitivity, we conducted nested PCR. However, it did not enhance the sensitivity.

We also evaluated detection limits of *A. fumigatus* and *C. albicans* DNA in serum. Extracted fungal DNA ranging from 10 ng to 10 fg was separately added to 200 μl of rabbit serum. When 1 μl of the serum sample was used directly as template for PCR, the detection limit was 5 fg per 1 μl of serum. After nested PCR, the minimal amount of DNA required for fungal identification decreased to 0.05 fg per 1 μl of serum (Fig. 2b).

### Identification of the Infected Fungi from Mice

Blood samples from infected mice were tested in place of actual human clinical samples. After 1 h of fungal infection, blood was collected and used directly for PCR amplification. Because the first-round PCR did not yield enough amplicon, nested PCR was performed to increase the labeled amplicon, making it possible to detect inoculated fungi in the blood from the infected mouse by microarray. At this moment, the CFU of *A. fumigatus* and FSSC remained in blood stream were 500 ± 50 (colonies/ml) and 230 ± 30 (colonies/ml), respectively. This detection level is consistent with the result of sensitivity test using rabbit whole blood spiked with fungal cells.

### Identification of the Fungi from Blood Culture Sample

Blood from a patient was cultivated in a blood culture bottle for 7 days; one culture positive and one negative sample were subjected to microarray analysis. Samples from blood culture bottle were used directly as a template for nested PCR amplification. The microarray result was consistent with the identification made by the Chiba University Hospital clinical laboratory.

### Isothermal Sample DNA Amplification

**Fig. 3:**
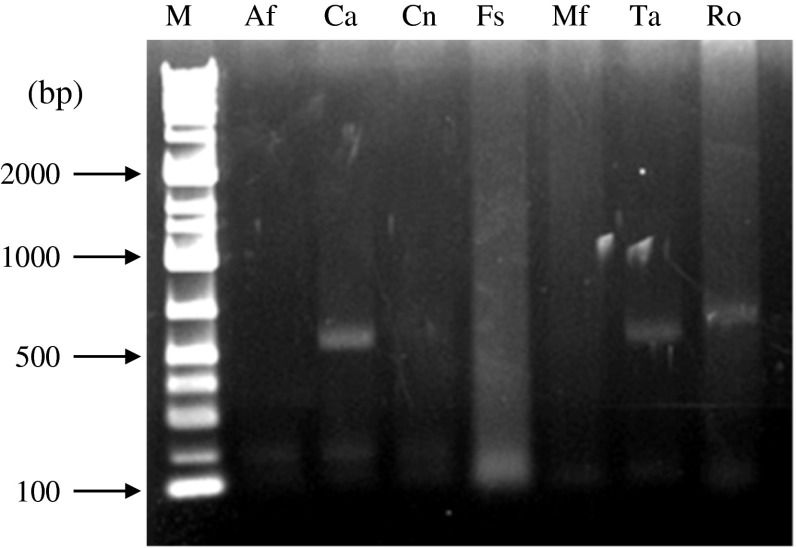
Isothermal amplification. Agarose gel electrophoresis of fungal DNA obtained by isothermal amplification using TwistAmp Basic kit. *Lanes*: M, molecular marker; Af, *A. fumigatus*; Ca, *C. albicans*; Cn, *Cryptococcus neoformans*; Fs, FSSC; Mf, *Malassezia furfur*; Ta, *T. asahii*; Ro, *Rhizopus oryzae*

Because PCR amplification requires a thermal cycler, which is not always available, in small hospitals, or in less developed regions, we attempted to carry out isothermal amplification for biotin labeling of sample DNA using RPA technique [13]. The RPA cycle was performed using 1.5 μl cell suspensions of various fungi as template (Fig. 3). If amplification reaction was successful, the microarray system we developed gave correct identification results in all of the tested samples, even though several amplification bands were observed in some samples.

## Discussion

Fungal infections cause severe morbidity and mortality in immunocompromised patients. Early start of proper treatment is crucial point to achieve better outcome in these patients. Because sensitivity to antifungal drugs differs among different fungal species, identification of the causative fungal agent is important for proper treatment. Rapid detection and identification of pathogens are therefore key points for diagnosis.

Recently, microarray methods have been developed for the identification of a variety of pathogens, including viruses [17], bacteria [4, 8], and fungi [5–7]. These methods are powerful tools to simultaneously detect multiple pathogens. In this study, we developed an easy-to-use, rapid and inexpensive microarray method utilizing biotin-conjugated HRP and color development of the substrate for signal detection. In addition to making the detection system straightforward, we immobilize DNA probes to ordinary plastic slides without any surface modification using UV irradiation via the poly-T anchor of the capture probes [14]. This immobilization system allowed us to use inexpensive, mass-produced, and commercially available ordinary plastic slides as the DNA microarray substrate.

For our DNA microarray, we selected ITS regions of fungal rRNA genes as target because the ITS sequence have been widely used to identify fungi. Although it was difficult to design species-specific probes for some of the fungal genera (*Alternaria* sp., *Rhizomucor* sp., *Mucor* sp., *Trichosporon* sp.), pathogenic species of these genera have similar MIC values against several antifungal drugs, making designing genus-specific probes useful [18–21]. To our knowledge, the number of fungal species/genera that could be identified by our array system, 42 species from 24 genera, is the largest among microarray identification systems reported to date [5–7]. And the number of identifiable fungal species can be further increased depending on demand of clinical use. Considering the increasing incidence of infectious diseases caused by fungi, this microarray system, which can be used to identify a variety of fungi simultaneously, has great potential.

The sensitivity of our microarray system was evaluated using whole blood spiked with a certain number of fungal cells and serum with fungal DNA. When 1 μl of either sample was used directly as the PCR template, the limit of the detection was 10^3^ cells/ml for blood, and 5 pg/ml of DNA for serum. Nested PCR increased the sensitivity to 50 fg/ml of DNA in serum, but no change in sensitivity was observed in the blood sample. According to calculations, in the 10^3^ cells/ml blood sample, 1 μl of template contains 1 cell, so in case of lower concentration sample, 1 μl of template does not contain any cells. That is why the amplification of 10^2^ cells/ml sample was not constant and sensitivity could not increase by nested PCR. However, DNA extraction or concentration of cells from larger volume of blood or serum sample will have a possibility to decrease detection limit.

When we tested the sample from an infected mouse model and blood culture, it was difficult to get enough intensity in microarray detection with only one step of PCR amplification. This indicated that the amount of DNA in the actual clinical samples is smaller than the detection limit of our microarray system. Nested PCR, however, increased the sensitivity of amplification, and the nested PCR samples successfully gave the expected diagnostic results.

The PCR step in conventional microarray systems has remained as a crucial barrier for wide use in laboratories or hospitals not equipped with a PCR machine. In the present work, we adopted isothermal amplification of DNA samples using the RPA technique as an alternative to PCR to solve this problem. Although, the RPA technique has been used for rapid identification of viruses and bacteria [13], this is the first report of fungal DNA amplification by the RPA technique directly from a fungal cell suspension within 1 h. However, in the present study, the RPA method was found to be less sensitive than the conventional PCR technique. Further optimization of sample preparation and RPA conditions are expected to yield improved results.

In conclusion, we were able to establish a rapid microarray system that can specifically identify a variety of fungal pathogens. Furthermore, we demonstrated that the ABC method could yield enough sensitivity to detect signals from clinical samples, providing an alternative to expensive fluorescence-scanning methods. We also demonstrated that the isothermal amplification technique in combination with this array system has high potential for future applications, such as for bedside diagnosis. This type of assay technique enables simultaneous identification of several agents in a few, relatively simple steps and therefore will become a useful tool in the identification of a wide range of both pathogenic and nonpathogenic microorganisms.

## Electronic supplementary material

Below is the link to the electronic supplementary material.

Supplementary material 1 (XLSX 23 kb)

Supplementary material 2 (PDF 51 kb)
