# Supplementary material for: Identification of Fungal Pathogens by Visible Microarray System in Combination with Isothermal Gene Amplification
Source: Mycopathologia. 2014 Jun 22;178(1):11–26. doi: 10.1007/s11046-014-9756-2 (PMC4098066; doi:10.1007/s11046-014-9756-2)
Supplement: Supplementary file 2 — Supplementary material 2 (PDF 51 kb) [file 11046_2014_9756_MOESM2_ESM.pdf]

TableS2 Reference sequences used to make allignment

Genus	Referred sequence number	Referred sequence number of objective	Objectibe fungi	Example accession No.
Absidia	57	12	*Absidia corymbifera*	FJ345350
Alternaria	42	-	*Alternaria alternata*	FJ717729
Aspergillus	110	15	*Aspergillus flavus*	HQ340110
		30	*Aspergillus fumigatus*	EF669999
		22	*Aspergillus nidulans*	AB248971
		18	*Aspergillus niger*	HQ607993
		15	*Aspergillus terreus*	AB369899
Blastomyces	9	6	*Blastomyces dermatitidis*	U18364
Candida	140	33	*Candida albicans*	HM016890
		20	*Candida dubliniensis*	AB369916
		12	*Candida famata*	HE681104
		18	*Candida glabrata*	AB467297
		8	*Candida guilliermondii*	AB369917
		6	*Candida kefyr*	GU256755
		5	*Candida krusei*	AY939808
		7	*Candida lusitaniae*	AY174102
		13	*Candida parapsilosis*	AB109231
		4	*Candida rugosa*	GU144663
		9	*Candida tropicalis*	AB467289
		5	*Candida zeylanoides*	AB278160
Coccidioides	8	5	*Coccidioides posadasii*	AB232896
Cryptococcus	56	20	*Cryptococcus gattii*	FJ534877
		13	*Cryptococcus neoformans*	CP003821
Cunninghamella	11	6	*Cunninghamella bertholletiae*	AF254931
Epidermophyton	7	4	*Epidermophyton floccosum*	AY213646
Exophiala	21	21	*Exophiala dermatitidis*	AF147495
Fusarium	111	-	*Fusarium oxysporum*	HQ148098
		64	*Fusarium solani (Fusarium solani complex: FSS*	AB551665
Histoplasma	10	8	*Histoplasma capsulatum*	AB071821
Malassezia	24	8	*Malassezia furfur*	AB105151
Microsporum	28	6	*Microsporum canis*	AB193669
		5	*Microsporus gypseum*	AB193671
Mucor	32	-	*Mucor hiemalis*	AY243951
Paracoccidioides	4	4	*Paracoccidioides brasiliensis*	AB304448
Penicillium	30	8	*Penicillium marneffei*	AB353919
Pichia	35	2	*Pichia anomala*	KC568565
		3	*Pichia norvegensis*	AB278169
Rhizomucor	12	-	*Rhizomucor miehei*	HM999960
		-	*Rhizomucor pusillus*	AB369914

Rhizopus	40	13	*Rhizopus microsporus*	DQ641315
		5	*Rhizopus oryzae*	DQ641354
Rhodotorula	39	7	*Rhodotorula mucilaginosa*	AF444738
Scedosporium	35	10	*Scedosporium prolificans*	EF568078
Sporothrix	30	7	*Sporothrix schenckii*	AB089139
Trichophyton	42	11	*Trichophyton rubrum*	AB194245
		10	*Trichophyton mentagrophytes*	JQ928411
		8	*Trichophyton tonsurans*	AB220044
Trichosporon	35	11	*Trichosporon asahii*	AB369919
		-	*Trichosporon asteroides*	FJ943421
		9	*Trichosporon cutaneum*	AB018020
